# Effects of Black Soldier Fly Larvae as Replacement of Soybean Meal on the Performance, Meat Quality, and Health Status in Broilers

**DOI:** 10.3390/vetsci13030282

**Published:** 2026-03-18

**Authors:** Ahmed A. A. Abdel-Wareth, Md Salahuddin, Prantic K. Goswami, Cassandra D. Gray, Adrian M. W. Aviña, Abigail Osei-Akoto, Trahmilla Carr, Alejandro Argueta, Lea Ann Kinman, Jayant Lohakare

**Affiliations:** 1 Poultry Center, Cooperative Agricultural Research Center, College of Agriculture, Food and Natural Resources, Prairie View A&M University, Prairie View, TX 77446, USA; aahabdelwareth@pvamu.edu (A.A.A.A.-W.); mdsalahuddin@pvamu.edu (M.S.); pgoswami@pvamu.edu (P.K.G.); cdgray@pvamu.edu (C.D.G.); amavina@pvamu.edu (A.M.W.A.); aoseiakoto@pvamu.edu (A.O.-A.); tcarr21@pvamu.edu (T.C.); alargueta@pvamu.edu (A.A.); 2 Department of Animal and Poultry Production, Faculty of Agriculture, Qena University, Qena 83523, Egypt; 3 Meat Science Center, Cooperative Agricultural Research Center, College of Agriculture, Food and Natural Resources, Prairie View A&M University, Prairie View, TX 77446, USA; lakinman@pvamu.edu

**Keywords:** broiler, black soldier fly larvae, soybean meal replacement, growth performance, carcass traits, blood biochemistry

## Abstract

This study investigated the potential of black soldier fly larvae (BSFL) meal to replace soybean meal in broiler diets by evaluating growth performance, carcass traits, meat quality, and blood biochemical responses. A total of 160 ten-day old Ross 708 chicks (216.74 ± 0.74, g) were randomly assigned to four dietary treatments containing 0%, 20%, 40%, or 60% BSFL meal replacing soybean meal on a 100% equivalent basis, respectively, and evaluated during the starter (10–21 days), grower (21–42 days), and overall (10–42 days) phases. Carcass characteristics, meat color, and blood biochemistry were assessed on day 42. Data was analyzed using polynomial (linear and quadratic) contrasts. Increasing dietary BSFL levels resulted in significant reductions in body weight, average daily gain, and feed intake, while the feed conversion ratio increased linearly. Carcass yield decreased to higher inclusion levels, accompanied by a marked increase in gizzard weight. Meat color (L*, a*, b*) remained largely unchanged across treatments. Blood biochemical analysis revealed linear and quadratic shifts in key metabolites, enzymes, and electrolytes, including reductions in aspartate aminotransferase, bilirubin, and creatine phosphokinase, as well as altered calcium and phosphorus concentrations. Overall, BSFL meal inclusion as 20% replacement improved growth performance and stimulated beneficial lipid and protein metabolism adaptations in broilers.

## Simple Summary

Soybean meal is the primary protein source in poultry diets, but its rising price and environmental impact are driving interest in more sustainable substitutes. Black soldier fly larvae meal offers a promising option because it is high in protein and can be produced efficiently using low-value organic substrates. In this study, Black soldier fly larvae meal was included in broiler diets at incremental levels to evaluate its potential to replace soybean meal and to assess its effects on growth performance, feed efficiency, carcass traits, meat quality, and key blood biochemical indicators. The results showed that low to moderate replacement levels of soybean meal with Black soldier fly larvae meal supported normal growth, physiological health, and meat quality. However, higher replacement levels negatively affected weight gain and feed conversion. These findings suggest that Black soldier fly larvae meal can partially replace soybean meal when incorporated at appropriate levels.

## 1. Introduction

The need for sustainable animal nutrition is a major focus in veterinary and animal sciences as livestock production systems now must find ways to support animal health and productivity while also ensuring food security and protecting the environment. Global food demand is projected to increase by approximately 35–56% by 2050, due to population growth and a global shift toward animal-based proteins, placing substantial pressure on current agricultural systems [1]. According to the OECD–FAO Agricultural Outlook, poultry meat is expected to contribute a major share of this animal protein consumption because of its affordability, production efficiency, and broad consumer acceptance [2]. As results, poultry production represents a critical sector for meeting rising global protein needs while maintaining environmental sustainability. Feed formulation plays a central role in poultry health and production efficiency, with feed costs representing up to 70% of total production expenditures [3]. Protein sources, particularly soybean meal (SBM), dominate poultry diets due to their high crude protein content and favorable amino acid profile. However, the extensive cultivation and international trade of SBM are associated with environmental concerns, including deforestation, biodiversity loss, greenhouse gas emissions, and competition with the human food and biofuel industries [4]. These economic and ecological challenges have intensified interest in alternative protein sources that are nutritionally adequate, cost-effective, and environmentally friendly. The growing environmental and economic pressures associated with conventional protein sources, particularly soybean meal, have intensified the search for sustainable and efficient alternatives. Insects have emerged as promising candidates due to their ability to convert low-value organic substrates into high-quality biomass with a much smaller environmental footprint. Among these options, black soldier fly larvae (BSFL; *Hermetia illucens*) stand out as one of the most scalable and environmentally sustainable protein sources available [5]. BSFL are characterized by high protein levels, a balanced essential amino acid profile, and appreciable lipid and mineral concentrations, supporting their suitability for monogastric animal nutrition [6,7]. Importantly, BSFL can efficiently convert a wide range of organic substrates, including agricultural by-products, food waste, and manure into high-quality biomass, thereby reducing waste and enabling nutrient recycling [8,9]. This capacity aligns BSFL production with circular economic principles and enhances its relevance within sustainable livestock systems. Recent advances in BSFL production technologies have improved larval yield, nutritional consistency, and scalability across diverse production environments. Optimization of rearing substrates and environmental conditions has been demonstrated in both tropical and subtropical regions, highlighting the feasibility of BSFL production even in resource-limited settings [10,11]. Moreover, large-scale BSFL protein production is rapidly expanding worldwide, with insect farming increasingly integrated into sustainable feed supply chains to reduce reliance on conventional protein sources and enhance circular economy practices [12]. The global adaptability of *H. illucens* is further supported by population genetic studies showing strong dispersal capacity and resilience across climatic zones [13], although climate change may influence its future geographic distribution [14]. Early studies demonstrated the successful replacement of conventional protein sources with insect meals in poultry diets, including BSFL meal in broiler diets [15]. Comprehensive evaluations have since confirmed the technical feasibility of insects as feed ingredients, emphasizing their digestibility, palatability, and nutritional value [4]. Beyond feed applications, BSFL biomass has also been explored for energy production, such as biodiesel extraction from larval lipids, further enhancing its multifunctional role in sustainable bioeconomy systems [16]. Despite the growing body of research, the nutritional application of BSFL in broiler diets remains associated with variable and sometimes contradictory outcomes. Recent reviews indicate that low levels of BSFL inclusion, typically around 10% during standard broiler grow-out periods (approximately 10–35 days), generally maintain or even enhance growth performance and feed efficiency. In contrast, near-complete replacement of soybean meal, particularly when full-fat BSFL is used, has consistently been associated with reductions in body weight gain and deterioration in feed conversion, emphasizing the importance of both inclusion level and larval fat content in determining nutritional outcomes [6,7]. These divergent responses have been attributed to factors such as chitin content in the larval exoskeleton, variability in amino acid digestibility, lipid composition, imbalances in mineral ratios, particularly calcium and phosphorus, differences in larval processing methods, and rearing substrates [5,7]. Such inconsistencies show the need for controlled, dose–response studies to clarify optimal inclusion levels and underlying physiological mechanisms. From a veterinary and physiological perspective, limited attention has been given to the effects of graded BSFL inclusion on carcass characteristics, meat quality, and systemic health indicators in broilers. Blood biochemical profiles offer critical insights into metabolic regulation and organ function but are often overlooked in insect-based feeding trials. Recent reviews showed the need to integrate growth performance with health indicators to ensure safe and effective use of BSFL in poultry diets [5]. We hypothesized that moderate BSFL inclusion, defined as 20–40% replacement of soybean meal, would maintain growth performance and physiological balance, whereas higher inclusion, defined as 60% replacement, could alter growth and metabolic responses. Accordingly, this study evaluated the effects of graded BSFL inclusion (0%, 20%, 40%, and 60% SBM replacement) on growth performance, carcass traits, meat quality, and blood biochemistry in broilers. The findings aim to provide evidence-based guidance on the potential and limitations of black soldier fly larvae as a sustainable protein source in poultry nutrition.

## 2. Materials and Methods

The study was conducted at the Poultry Center in Prairie View A&M University (PVAMU), Prairie View, TX, USA. All animal handling and experimental procedures adhered to the Animal Welfare Act (AWA) and Animal Welfare Regulations (AWRs) established by the United States Department of Agriculture (USDA), as well as the Guide for the Care and Use of Laboratory Animals [17,18]. Ethical approval was obtained from the Institutional Animal Care and Use Committee (IACUC) of PVAMU under protocol number AUP #2022-045.

### 2.1. Experimental Design and Diets

This study was conducted to evaluate the effects of partially replacing soybean meal with graded levels of Black Soldier Fly Larvae (BSFL) meal on broiler growth performance, carcass characteristics, meat quality, and blood biochemical parameters. A total of 200 one-day-old male Ross 708 broiler chicks (average body weight: 42.5 ± 2.1 g) were obtained from a commercial hatchery. To ensure uniform early growth and to minimize stress-related variability, all chicks received a control starter diet from day 1 to day 10. At 10 days of age (average body weight: 216.74 ± 0.74 g), birds were randomly allocated to four dietary treatments containing 0% (control), 20%, 40%, or 60% BSFL meal, replacing soybean meal on a 100% equivalent protein basis. These inclusion levels were selected based on previously published studies [6,7]. Each treatment consisted of eight replicates with five birds per replicate. Birds were housed under standard management conditions in floor pens with wood-shaving bedding, controlled temperature, and continuous access to feed and water. From day 10 to day 42, the experimental diets were provided in two phases: a starter phase (days 10–21) and a grower phase (days 21–42). Using a common diet during the first 10 days also allowed chicks to adapt to the housing environment and ensured that the treatment effects reflected dietary responses during the main growing period. The experimental diets were formulated to meet or exceed the Ross 708 Broiler Nutrient Requirements Guidelines [19] for the starter (Table 1) and grower (Table 2) phases, based on the total amino acid content of the ingredients. Whole dried BSFL was used as a partial replacement for soybean meal, and standardized ileal digestible (SID) amino acid values were not specifically applied, although all diets provided sufficient amino acids to support normal growth and performance under the experimental conditions. Requirements for each phase were balanced using synthetic amino acids (L-lysine, DL-methionine, and L-threonine). Specifically, starter diets (Table 1) supplied Lysine (%) of 1.207, 1.170, 1.141, and 1.108 for the 0%, 20%, 40%, and 60% BSFL treatments, respectively; grower diets (Table 2) supplied Lysine (%) of 1.081, 1.049, 1.019, and 0.989 for the corresponding treatments. Diets were formulated to be near-isoenergetic; however, a small, systematic increase in calculated ME occurred with higher BSFL inclusion (starter: 12.45, 12.54, 12.61, 12.66 MJ/kg; grower: 13.060, 13.120, 13.188, 13.221 MJ/kg for 0%, 20%, 40%, and 60% BSFL, respectively).

### 2.2. Growth Performance

Growth performance was assessed by recording body weight at days 10, 21, and 42. Daily weight gain, feed intake, and feed conversion ratio (FCR) were calculated for the starter (10–21 days), grower (21–42 days), and overall (10–42 days) periods. Mortality was monitored daily and used to adjust performance calculations.

### 2.3. Black Soldier Fly Larvae Source and Composition

The BSFL used in this study was sourced from EnviroFlight, LLC (EnviroBug^®^, Darling Ingredients), produced in Apex, NC, USA. The product consisted of whole dried larvae, classified by AAFCO [20] as a novel protein and fat source for poultry feed. The BSFL ingredient used was whole dried larvae, and its nutrient composition is presented in Table 3. BSFL were fed distillers grain and stillage from the bourbon industry and bakery by-products. Whole dried black soldier fly larvae (BSFL) were used instead of defatted or ground meal to reflect practical feed production conditions, as whole dried larvae are commonly incorporated into commercial poultry diets in a cost-effective manner. This approach also allowed evaluation of the combined effects of the naturally occurring chitin and lipid fractions present in whole larvae, which may influence nutrient digestibility and gastrointestinal function.

### 2.4. Carcass Criteria

At the end of the trial (day 42), two birds per replicate were randomly selected and slaughtered in accordance with animal welfare guidelines, following humane procedures. After evisceration, carcass yield was calculated as the percentage of eviscerated carcass weight relative to live body weight. Abdominal fat and internal organs, including the liver, gizzard, heart, spleen, pancreas, small intestine, and cecum, were excised, weighed individually, and expressed as a percentage of live body weight to assess organ development and fat deposition. For meat quality assessment, breast muscle samples (pectoralis major) were collected immediately after slaughter. Meat color was determined using a calibrated colorimeter (CR-400 HEAD Package W/NX2 LITE, Konica Minolta Business Solutions U.S.A., Inc., Ramsey, NJ, USA) based on the CIE color system, which measures three parameters: lightness (L*), redness (a*), and yellowness (b*). The instrument was standardized before each measurement using a white calibration plate to ensure accuracy.

### 2.5. Blood Biochemical Analysis

Blood samples were collected from the brachial vein on day 42 into 10 mL tubes immediately after slaughtering the birds. Serum was separated by centrifugation at 3000× *g* for 15 min at 4 °C using a Sorvall ST Plus centrifuge (Thermo Electron LED GmbH, Langenselbold, Germany) and stored at −20 °C until further analysis to maintain sample integrity. Serum biochemical parameters were analyzed at the Arkansas Veterinary Diagnostic Laboratory using the Avian/Reptile Chemistry Panel on a Dimension EXL 200 automated chemistry analyzer (Siemens Healthcare Diagnostics Inc., Newark, DE, USA).

Biochemical measurements included a comprehensive panel of parameters to assess the birds’ physiological status. Electrolytes measured included sodium, potassium, and chloride. Metabolite analysis covered glucose, cholesterol, uric acid, blood urea nitrogen (BUN), total protein, and total bilirubin. Mineral concentrations of calcium and phosphorus were also determined. Enzymatic activities were assessed for aspartate aminotransferase (AST), gamma-glutamyl transferase (GGT), alkaline phosphatase (ALP), and creatine phosphokinase (CPK). Additionally, serum osmolality was measured to assess osmotic balance. Commercial assay kits were used according to manufacturer instructions, and strict quality control procedures were followed to ensure accuracy and reproducibility. Serum osmolality was measured using a calibrated osmometer integrated into the analyzer system. In the laboratory, all analytes were measured using Siemens Dimension Flex reagent cartridges (e.g., DF33B Creatinine Flex Reagent Cartridge, Newark, DE, USA) specifically designed for the Dimension EXL analyzer. All procedures followed the manufacturer’s validated operating protocols as described in the Siemens Operator’s Guide. This standardized diagnostic platform ensured reliable quantification of electrolytes, metabolites, minerals, and enzyme activities, enabling a robust assessment of physiological responses to dietary treatments.

### 2.6. Statistical Analysis

Data were analyzed using the General Linear Model (GLM) procedure in SAS version 9.4, SAS Institute Inc., Cary, NC, USA [21] under a completely randomized design. Dietary treatment was considered a fixed effect in the model. The statistical model applied was:

$$Y_{ij}=\mu +T_{i}+\varepsilon_{ij}$$

where $Y_{ij}$ represents the observed response variable, μ is the overall mean, $T_{i}$ is the fixed effect of the ith treatment (dietary BSFL inclusion level), and $\varepsilon_{ij}$ is the random error term associated with the observation.

Orthogonal polynomial contrasts were used to evaluate linear and quadratic dose-dependent responses to increasing BSFL inclusion levels. Statistical significance was accepted for *p*-values below 0.05, with values < 0.001 reported as 0.001. Results are presented as means ± standard error of the mean (SEM). All figures were generated using GraphPad Prism version 9 [22] for clear visualization of treatment effects.

## 3. Results

### 3.1. Growth Performance

Replacing soybean meal with BSFL in broiler diets led to clear linear changes in most growth metrics (Table 4). Body weight decreased linearly at both 21 and 42 days as BSFL inclusion increased (*p* < 0.001). Similarly, daily body weight gain declined significantly across all growth phases (10–21, 21–42, and 10–42 days; *p* < 0.001), indicating a consistent reduction in growth rate at higher BSFL levels. Feed intake also decreased linearly over the entire 10–42-day period (*p* = 0.026). During the starter phase (10–21 days), a significant quadratic effect was observed (*p* = 0.025), suggesting a non-linear response at moderate inclusion levels. The feed conversion ratio (FCR) increased linearly with increasing BSFL inclusion across all phases (*p* ≤ 0.002), with an additional quadratic effect observed during the 10–21-day period (*p* = 0.018), suggesting a possible inflection point at intermediate BSFL levels. Overall, replacing soybean meal with BSFL led to a progressive decline in growth performance, particularly at inclusion rates above 20%. No mortality was recorded in any treatment throughout the experimental period.

### 3.2. Carcass Criteria and Meat Quality

Replacing soybean meal with increasing levels of BSFL in broiler diets had notable effects on certain carcass traits, while the impact on meat quality parameters was generally limited (Table 5). Dressing percentage decreased overall with increasing BSFL inclusion (linear, *p* = 0.014), reaching the lowest value at 40% inclusion, with a quadratic trend also observed (*p* = 0.088), indicating some recovery at the highest inclusion level (60%). A quadratic trend was also observed (*p* = 0.088), indicating some variability in the response at intermediate inclusion levels. Among internal organs, gizzard weight increased significantly in both a linear (*p* < 0.001) and quadratic (*p* = 0.025) manner, suggesting possible changes in digestive activity or adaptation to the diet. Other organs, including the liver, heart, spleen, pancreas, small intestine, and cecum, were not significantly affected by BSFL inclusion. Although abdominal fat showed a decrease with increasing BSFL levels, the effect was not statistically significant (*p* = 0.179). Regarding meat quality, no significant linear or quadratic responses were found for lightness (L*), redness (a*), or yellowness (b*), though a trend toward reduced yellowness at the highest BSFL level approached significance (*p* = 0.066). Overall, the inclusion of BSFL altered carcass yield and gizzard development but did not significantly affect most meat quality attributes.

### 3.3. Blood Biochemical Parameters

#### 3.3.1. Electrolytes and Osmoregulation

**Figure 1:**
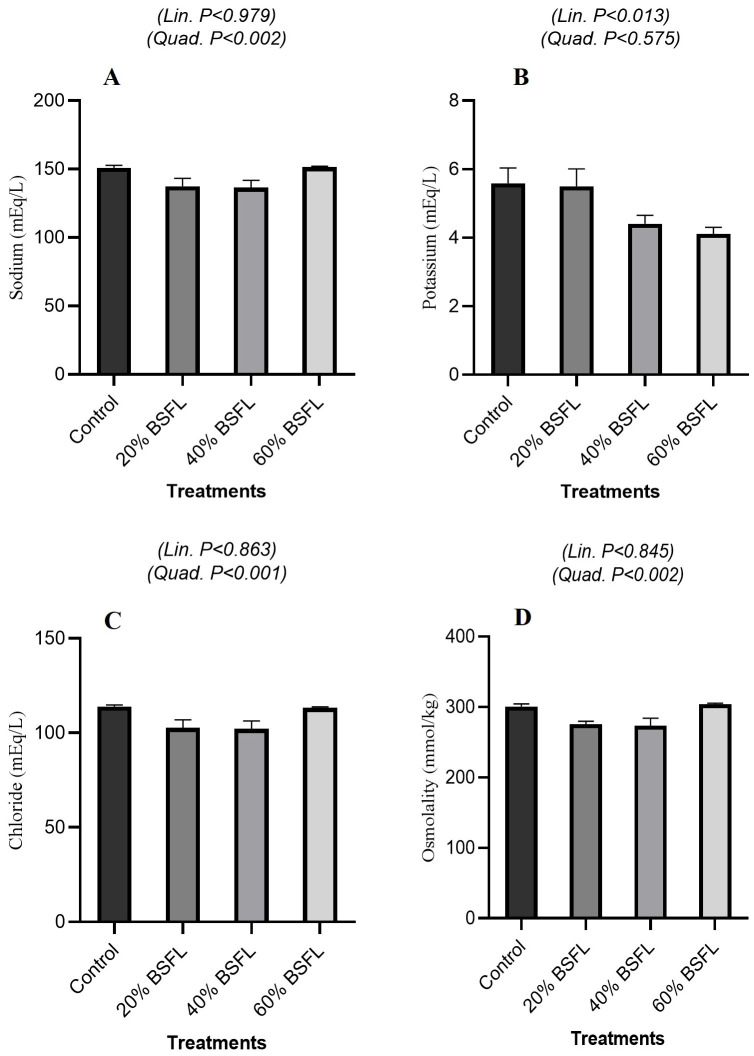
Serum Electrolytes and Osmoregulation in Broiler Fed Black Soldier Fly Larvae (BSFL). Effect of graded dietary BSFL inclusion on serum sodium (**A**), potassium (**B**), chloride (**C**), and osmolality (**D**) of broiler chickens. Data are presented as means ± SEM. Linear and quadratic trend *p*-values are shown within each panel.

As shown in Figure 1A, serum sodium concentrations were not significantly affected by BSFL inclusion in a linear manner (*p* = 0.979) but exhibited a significant quadratic response (*p* = 0.002). Similarly, Figure 1C shows that chloride concentrations remained stable across treatments (linear trend: *p* = 0.863) yet demonstrated a strong quadratic effect (*p* < 0.001). In contrast, Figure 1B indicates that potassium levels decreased significantly with increasing BSFL inclusion (linear trend: *p* = 0.013), while the quadratic effect was not significant (*p* = 0.575). Finally, Figure 1D illustrates that serum osmolality was generally unaffected in a linear fashion (*p* = 0.845) but showed a significant quadratic trend (*p* = 0.002).

#### 3.3.2. Hepatic Function and Cholestasis

**Figure 2:**
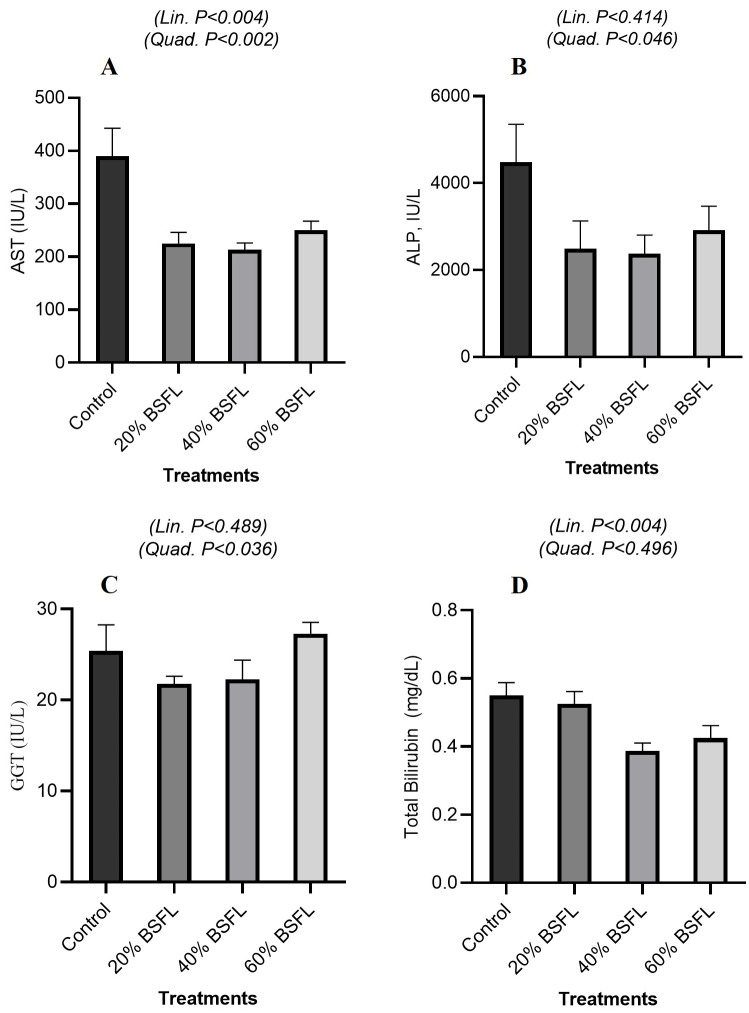
Hepatic Function and Cholestasis in Broiler Fed Black Soldier Fly Larvae (BSFL). Effect of graded dietary BSFL inclusion on hepatic biomarkers: (**A**) aspartate aminotransferase (AST), (**B**) alkaline phosphatase (ALP), (**C**) gamma-glutamyl transferase (GGT), and (**D**) total bilirubin of broiler chickens. Data are presented as means ± SEM. Linear and quadratic trend *p*-values are indicated within each panel.

As shown in Figure 2A, serum AST activity decreased overall with increasing BSFL inclusion, with a significant linear effect (*p* = 0.004) and a quadratic trend (*p* = 0.002), reflecting a greater reduction at moderate inclusion levels (20–40%) followed by a slight increase at 60% BSFL. In Figure 2B, ALP activity was less affected, with no significant linear trend (*p* = 0.414) but a marginal quadratic effect (*p* = 0.046). Similarly, Figure 2C shows that GGT activity remained relatively stable (linear *p* = 0.489), though a modest quadratic response was observed (*p* = 0.036). Finally, as shown in Figure 2D, serum total bilirubin decreased overall with increasing BSFL inclusion, exhibiting a significant linear effect (*p* = 0.004), while the quadratic effect was not significant (*p* = 0.496).

#### 3.3.3. Energy and Nitrogen Metabolism

**Figure 3:**
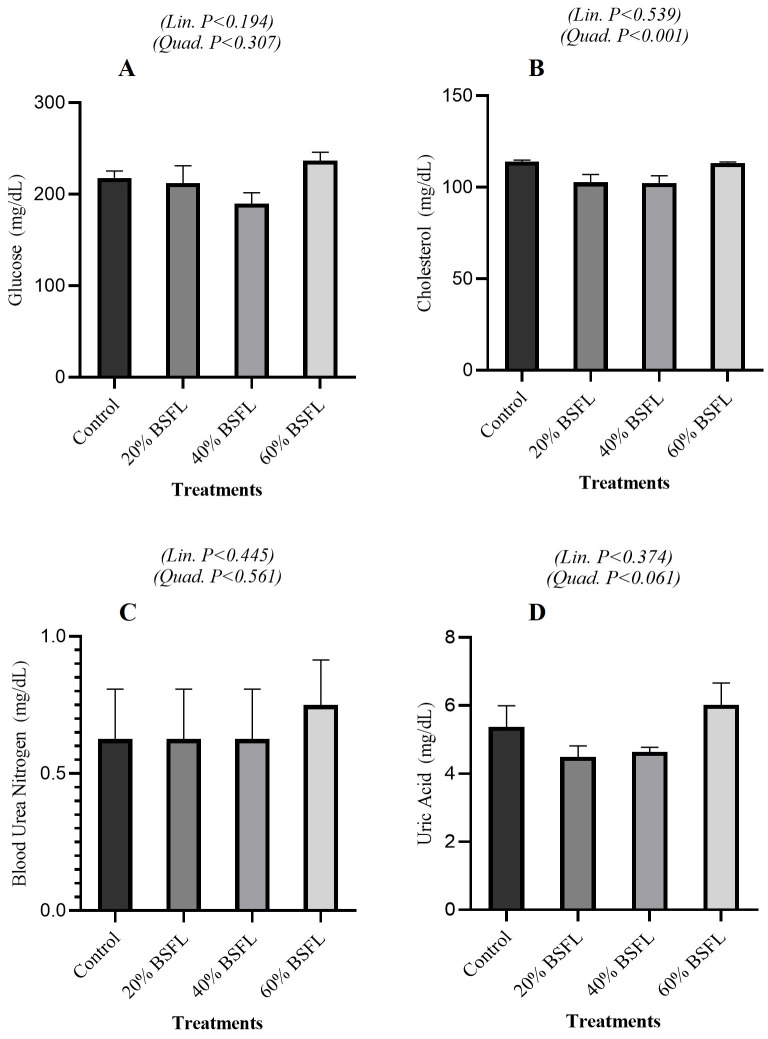
Energy and Nitrogen Metabolism in Broiler Fed Black Soldier Fly Larvae (BSFL). Effect of graded dietary BSFL inclusion on serum glucose (**A**), cholesterol (**B**), blood urea nitrogen (**C**), and uric acid (**D**) concentrations of broiler chickens. Data are presented as means ± SEM. Linear and quadratic trend *p*-values are indicated within each panel.

As shown in Figure 3A, serum glucose concentrations varied across treatments but exhibited no significant linear (*p* = 0.194) or quadratic (*p* = 0.307) trends. Similarly, Figure 3B shows that cholesterol levels were unaffected in a linear manner (*p* = 0.539), though a significant quadratic effect was observed (*p* < 0.001). In Figure 3C, blood urea nitrogen remained stable across BSFL inclusion levels (linear *p* = 0.445; quadratic *p* = 0.561). Finally, Figure 3D illustrates that uric acid concentrations did not differ significantly among treatments (linear *p* = 0.374; quadratic *p* = 0.061).

#### 3.3.4. Protein and Mineral Status

**Figure 4:**
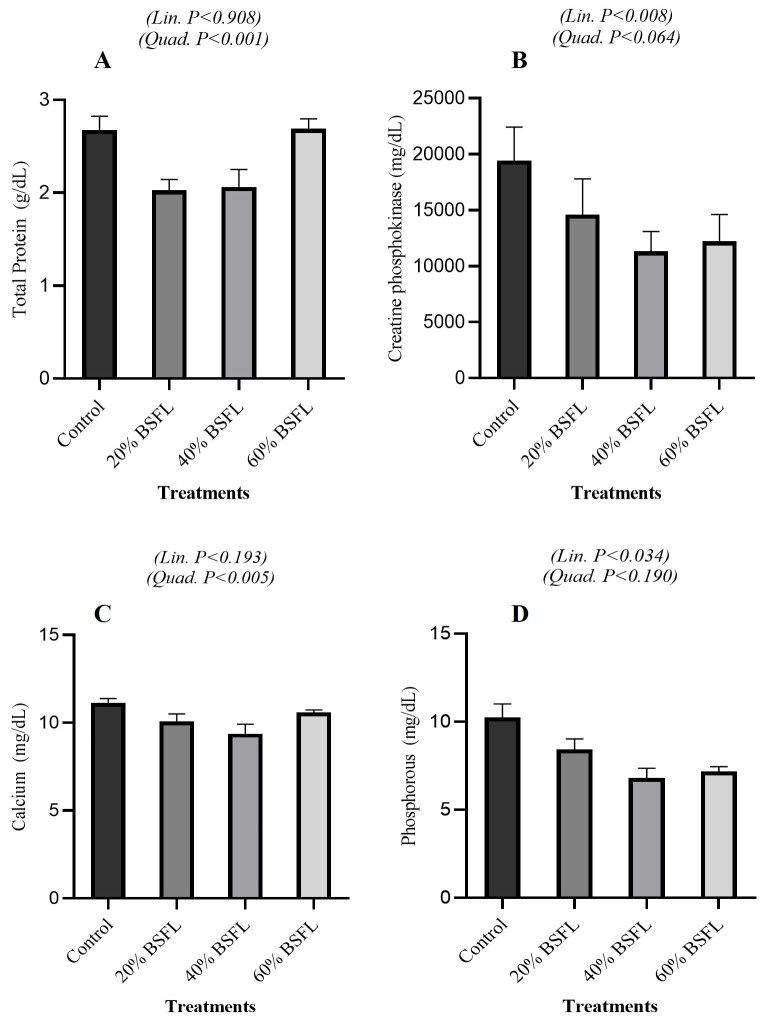
Protein and Mineral Status in Broiler Fed Black Soldier Fly Larvae (BSFL). Effect of graded dietary BSFL inclusion on total protein (**A**), creatine phosphokinase (**B**), calcium (**C**), and phosphorus (**D**) concentrations of broiler chickens. Data are presented as means ± SEM. Linear and quadratic trend *p*-values are indicated within each panel.

As shown in Figure 4A, total protein concentrations were unaffected in a linear manner (*p* = 0.908) but exhibited a significant quadratic trend (*p* = 0.001). In Figure 4B, creatine phosphokinase concentrations decreased significantly with increasing BSFL inclusion, showing a strong linear effect (*p* = 0.008) and a marginal quadratic response (*p* = 0.064).

Figure 4C shows that calcium levels remained stable in a linear fashion (*p* = 0.193) but displayed a significant quadratic effect (*p* = 0.005). Figure 4D shows that phosphorus concentrations decreased linearly with increasing BSFL inclusion (*p* = 0.034), whereas the quadratic effect was not significant (*p* = 0.190).

## 4. Discussion

Growth performance metrics were sensitive to increasing BSFL inclusion, with body weight and average daily gain declining linearly at both 21 and 42 days of age. The consistency of this trend across starter, grower, and overall periods suggests a sustained limitation on growth potential rather than a transient adaptation. Similarly, feed intake declined linearly throughout the experimental period, indicating that reduced nutrient intake directly contributed to impaired growth. Interestingly, quadratic responses for feed intake and FCR during the starter phase (10–21 days) suggest an early inflection point at intermediate BSFL inclusion levels. This pattern likely reflects an initial adaptive response of the immature digestive system at moderate BSFL inclusion. Birds can partially compensate via enhanced gizzard activity and prolonged digesta retention, improving mechanical breakdown of chitinous particles; however, as inclusion rises, protein embedded in the protein–chitin matrix reduces protease access and amino-acid availability, and the medium-chain fatty acid and saturated fatty acid-rich lipid profile (e.g., lauric acid) limits micelle formation and fat emulsification, lowering metabolizable energy and post-absorptive EAA supply [23,24]. Such age-dependent sensitivity is consistent with reports that younger broilers are particularly vulnerable to imbalances in essential amino acids, altered dietary structure, and shifts in energy partitioning when alternative proteins are introduced [23,24]. The worsening feed conversion (higher FCR) across phases indicates that reduced nutrient efficiency, rather than intake alone, drove performance losses, plausibly via greater gizzard workload (higher maintenance costs), lower lipid digestibility, and diminished amino-acid utilization at higher inclusion [23,24]. Collectively, these data show that replacing SBM with BSFL at ~20% of dietary protein results in a measurable growth penalty, a response observed across Ross, Cobb, and Arbor Acres genotypes [23,24,25]. Similar to Permana et al. [26], our findings indicate that moderate BSFL inclusion can maintain growth performance and feed conversion efficiency. However, at higher inclusion levels, excessive structural load and chitin content likely impaired nutrient digestibility, reflected in worsening FCR [26]. Low to moderate inclusion levels generally support acceptable growth and feed efficiency, whereas higher soybean meal replacement may reduce body weight gain and worsen FCR [6,7]. These differences may be explained by variation in chitin content, amino acid availability, lipid composition, mineral balance, particularly calcium and phosphorus, and differences in larval rearing and processing methods [5,7].

The linear decline in carcass yield with increasing BSFL inclusion indicates that reduced growth was reflected directly in a lower dressing percentage, rather than being offset by compensatory tissue deposition. Although a quadratic trend suggested partial stabilization at intermediate inclusion levels, this was insufficient to prevent overall yield reduction at higher doses. The lower carcass yield observed at 40% relative to 60% may reflect non-linear digestive adaptations at intermediate inclusion, wherein greater structural load from chitin-rich particles and altered diet physical characteristics transiently increase gizzard workload and prolong digesta retention, reducing the efficiency of carcass deposition before partial adaptation (e.g., more effective grinding/retention dynamics) develops at the highest inclusion—without fully restoring overall growth [25,27]. Consistent with this interpretation, the gizzard exhibited strong linear and quadratic increases in relative weight, indicating a pronounced physiological adaptation of the digestive system; this enlargement likely results from the greater mechanical effort required to process chitin-rich BSFL particles, which increase diet abrasiveness and stimulate gizzard musculature, while prolonged retention reflects the bird’s attempt to enhance grinding and nutrient extraction under higher structural load [28]. The literature reports BSFL chitin at ~5.6–6.7% DM, with variability by substrate and life stage [5,7,10,23]. At moderate levels, chitin (as an insoluble fiber) is associated with greater gizzard development, longer digesta retention, and enhanced digestive activity, supporting starch and lipid utilization [5,7]. To better explain the observed performance and metabolic responses, follow-up trials should incorporate gut histomorphology and microbiome profiling to determine whether BSFL alters mucosal architecture, microbial community structure, and fermentation end-products relevant to nutrient availability and health.

While enhanced gizzard development may improve digestive efficiency at low BSFL inclusion levels, excessive structural load at higher levels likely reduces nutrient availability and impairs feed efficiency, as reflected in the deteriorating FCR [26,28,29]. Other visceral organs, including the liver, pancreas, and intestines, remained unaffected, suggesting that BSFL inclusion may not induce overt hypertrophy or pathological stress in these organs. The non-significant reduction in abdominal fat suggests that energy intake limitations and altered lipid metabolism prevented excessive fat deposition, even at higher dietary fat levels from BSFL. Our findings agree with Permana et al. [26], who observed that BSFL meal inclusion at 7.5% and 15% significantly increased gizzard weight compared to the control diet, while carcass yield and composition remained unaffected at these moderate levels. This supports the hypothesis that chitin-rich BSFL particles and altered dietary structure stimulate gizzard musculature, enhance mechanical digestion, and potentially improve nutrient utilization when inclusion levels remain within practical limits [7,28]. A limitation of the present study is that meat quality assessment was restricted to color measurements, and parameters such as pH, drip loss, cooking loss, and shear force were not evaluated. Future studies should include a more comprehensive meat quality profile to fully characterize the effects of BSFL inclusion. BSFL inclusion had minimal impact on meat quality attributes, and the absence of significant changes in lightness, redness, or yellowness indicates that muscle pigmentation, myoglobin redox stability, and postmortem glycolytic metabolism were physiologically preserved. The near-significant reduction in yellowness at the highest inclusion level may be explained by reduced dietary carotenoid intake and shifts in lipid deposition, as BSFL contains fewer carotenoid precursors and a different fatty-acid profile than soybean-based diets; however, the overall stability of color parameters aligns with previous reports showing limited effects of BSFL on meat appearance at practical inclusion rates [27,30,31,32]. These findings suggest that BSFL-related reductions in performance do not extend to deterioration of consumer-relevant quality traits, reinforcing that the primary limitations of high BSFL inclusion arise from nutritional and digestive constraints rather than structural or pathological muscle alterations [7,26,28].

Serum electrolyte profiles demonstrated strong homeostatic regulation despite dietary changes. The quadratic fluctuations in sodium and chloride without linear trends likely reflect short-term compensatory adjustments of renal and respiratory buffering systems, rather than sustained electrolyte imbalance. The linear decline in potassium is consistent with the lower intrinsic potassium content of BSFL compared with potassium-rich soybean meal, leading to predictable shifts in dietary electrolyte supply. Importantly, serum osmolality remained stable, indicating that birds preserved osmotic balance through physiological regulation of water and ion fluxes, a response consistent with core principles of dietary electrolyte balance (DEB) whereby alterations in Na^+^, K^+^, and Cl^−^ ratios can be absorbed by homeostatic mechanisms without disrupting acid–base equilibrium as long as overall DEB remains within physiological thresholds [33,34,35]. Markers of hepatic function showed a consistent reduction in AST activity and total bilirubin with increasing BSFL inclusion, with both linear and quadratic effects. These responses indicate reduced hepatocellular enzyme leakage rather than hepatic stress or damage. ALP and GGT exhibited only modest quadratic responses, further supporting the absence of cholestatic or hepatotoxic effects. These findings align with previous studies reporting stable liver enzyme profiles in broilers fed BSFL-based diets [23,36] and suggest that BSFL inclusion, even at higher levels, does not compromise hepatic integrity when diets are nutritionally balanced.

Serum glucose, cholesterol, blood urea nitrogen, and uric acid concentrations remained largely unchanged across BSFL inclusion levels, with only quadratic variation observed for cholesterol. This indicates that systemic energy and nitrogen metabolism were preserved despite reduced growth performance. The lack of change in uric acid and urea nitrogen suggests that protein catabolism was not exacerbated at higher BSFL inclusion levels, supporting the interpretation that growth suppression was driven primarily due to lower nutrient availability and efficiency rather than increased protein catabolism [5,32]. Quadratic cholesterol responses likely reflect shifts in lipid absorption and transport associated with BSFL-derived fatty acids, particularly lauric acid, as reported previously [32,37]. Total serum protein exhibited a significant quadratic response without a linear trend, indicating maintained protein status with transient modulation at intermediate inclusion levels. The linear reduction in CPK activity suggests reduced muscle membrane leakage or altered muscle workload, potentially reflecting lower locomotor activity or reduced growth-related muscle turnover, as supported by Tellis et al. [38]. The quadratic response of calcium and linear decline in phosphorus are consistent with the high calcium and variable Ca:P ratio of BSFL (Table 3). Even when diets are formulated to meet requirements, these intrinsic mineral characteristics can influence circulating levels, emphasizing the importance of precise mineral formulation [39,40,41]. The experimental design provided sufficient precision for growth and carcass traits; however, several blood biochemical variables showed greater variability, which may have limited our ability to detect subtle treatment effects. Future studies should increase the number of sampled birds and/or perform a priori power analyses to enhance sensitivity. Taken together, the present results demonstrate that partially replacing soybean meal with BSFL results in a series of linear reductions in performance, accompanied by compensatory physiological adjustments. Growth suppression, reduced carcass yield, and pronounced gizzard hypertrophy were the principal consequences at higher inclusion levels, reflecting limitations in nutrient digestibility, amino-acid availability, and diet structure rather than pathological disturbances. In contrast, serum biochemical, hepatic, and electrolyte parameters remained largely within normal physiological ranges, indicating that the birds’ metabolic and homeostatic functions were preserved despite shifts in nutrient composition. These findings support the conclusion that the performance constraints associated with high BSFL inclusion are primarily nutritional and physicochemical in origin, not indicators of organ dysfunction or systemic distress. From a formulation standpoint and based on literature-informed practice rather than the present dose range, BSFL inclusion is best considered at moderate levels (≈5–12%) unless diets are extensively rebalanced for amino acids, dietary electrolyte balance, and mineral ratios [23,25,42]. Our findings showing performance penalties ≥ 20%, support this hypothesis, generating a range and underscore the need for targeted formulation when inclusion exceeds moderate levels. Strategies such as defatting BSFL, managing chitin content, and supplementing limiting amino acids remain critical for maximizing the productive value of BSFL while maintaining broiler performance and physiological health. Limitations and future work: An economic feasibility analysis comparing BSFL-based diets with conventional soybean meal diets was not performed here; given the practical importance of cost, processing (e.g., defatting), and market price variability, a dedicated cost-effectiveness assessment should be addressed in future studies alongside biological performance outcomes. Furthermore, a practical consideration is the potential batch-to-batch variability in BSFL nutrient composition, which can differ with rearing substrate, processing method, and larval age; such variability may influence diet consistency and should be evaluated in future work.

## 5. Conclusions

The present study demonstrates that replacing soybean meal with black soldier fly larvae (BSFL) at low- to moderate levels (20–40%) maintains growth performance, nutrient digestibility, physiological status, and meat quality in broiler chickens, whereas a high inclusion level (60%) negatively affects productive responses. These findings indicate that broilers can effectively utilize BSFL as a partial protein substitute at moderate inclusion levels when fed properly formulated diets. Therefore, BSFL can be recommended for partial soybean meal replacement up to 40%, while higher inclusion levels require further dietary optimization to prevent potential nutritional limitations and performance decline.

## Figures and Tables

**Table 1:** Ingredient and chemical composition of the starter experimental diets of broiler chicks.

Ingredients	Control	20% BSFL	40% BSFL	60% BSFL
Corn	55.000	55.000	55.000	55.000
Soybean meal 48%	30.000	24.000	18.000	12.000
Black soldier fly larvae (BSFL) 41.4% CP	0.000	6.000	12.000	18.000
Corn Gluten 60%	6.500	7.610	8.500	9.400
Soybean oil	4.110	3.000	2.110	1.210
Vitamin ^1^	0.250	0.250	0.250	0.250
Trace Minerals ^1^	0.050	0.050	0.050	0.050
Limestone	1.510	1.510	1.510	1.510
Mon-calcium phosphate	1.550	1.550	1.550	1.550
Salt	0.291	0.291	0.291	0.291
L-Lysine	0.244	0.244	0.244	0.244
DL-Methionine	0.341	0.341	0.341	0.341
L-Threonine	0.154	0.154	0.154	0.154
Total (kg)	100	100	100	100
Metabolizable energy (MJ/kg)	12.45	12.54	12.61	12.66
Crude protein (%)	23.25	23.28	23.19	23.16
Crude fiber%	2.325	2.603	2.810	2.990
Ether extract%	6.636	7.962	7.990	8.450
Calcium (%)	0.961	1.001	1.041	1.081
Phosphorus (%)	0.731	0.739	0.741	0.753
Lysine (%)	1.207	1.170	1.141	1.108
Methionine (%)	0.625	0.633	0.640	0.647

^1^ The vitamin–mineral premix contained the following levels per kilogram: 8,818,342 IU of vitamin A; 3,086,420 IU of vitamin D_3_; 36,742 IU of vitamin E; 13 mg of vitamin B_12_; 1177 mg of menadione; 4775 mg of riboflavin; 16,168 mg of D-pantothenic acid; 2350 mg of thiamine; 36,742 mg of niacin; 5732 mg of vitamin B_6_; 1398 mg of folic acid; 104,460 mg of choline; 441 mg of biotin; 120 mg of manganese (Mn); 1.4 mg of copper (Cu); 120 mg of zinc (Zn); 120 mg of iron (Fe); 0.5 mg of selenium (Se); and 800 mg of iodine (I). Control: Basal diet without Black Soldier Fly Larvae meal. 20% BSFL: Diet containing 20% Black Soldier Fly Larvae meal as a replacement for soybean meal. 40% BSFL: Diet containing 40% Black Soldier Fly Larvae meal as a replacement for soybean meal. 60% BSFL: Diet containing 60% Black Soldier Fly Larvae meal as a replacement for soybean meal.

**Table 2:** Ingredient and chemical composition of the grower experimental diets of broiler chicks.

Ingredients	Control	20% BSFL	40% BSFL	60% BSFL
Corn	57.000	57.000	57.000	57.000
SBM48%	28.000	22.400	16.800	11.200
Black soldier fly larvae (BSFL) 41.4% CP	0.000	5.600	11.200	16.800
Corn Gluten 60%	3.000	4.000	5.400	6.800
Soybean oil	7.939	6.939	5.539	4.139
Vitamin ^1^	0.250	0.250	0.250	0.250
Trace Minerals ^1^	0.050	0.050	0.050	0.050
Limestone	1.440	1.440	1.440	1.440
Mon-calcium phosphate	1.420	1.420	1.420	1.420
Salt	0.340	0.340	0.340	0.340
L-Lysine	0.194	0.194	0.194	0.194
DL-Methionine	0.282	0.282	0.282	0.282
L-Threonine	0.085	0.085	0.085	0.085
Total (kg)	100	100	100	100
Metabolizable energy (MJ/kg)	13.060	13.120	13.188	13.221
Crude Protein (%)	20.376	20.382	20.290	20.288
Crude fiber%	2.787	2.523	2.781	3.051
Ether extract%	11.720	11.721	11.891	12.483
Calcium (%)	0.897	0.935	0.974	1.013
Phosphorus (%)	0.681	0.689	0.697	0.706
Lysine (%)	1.081	1.049	1.019	0.989
Methionine (%)	0.552	0.559	0.567	0.575

^1^ The vitamin–mineral premix contained the following levels per kilogram: 8,818,342 IU of vitamin A; 3,086,420 IU of vitamin D_3_; 36,742 IU of vitamin E; 13 mg of vitamin B_12_; 1177 mg of menadione; 4775 mg of riboflavin; 16,168 mg of D-pantothenic acid; 2350 mg of thiamine; 36,742 mg of niacin; 5732 mg of vitamin B_6_; 1398 mg of folic acid; 104,460 mg of choline; 441 mg of biotin; 120 mg of manganese (Mn); 1.4 mg of copper (Cu); 120 mg of zinc (Zn); 120 mg of iron (Fe); 0.5 mg of selenium (Se); and 800 mg of iodine (I). Control: Basal diet without Black Soldier Fly Larvae meal. 20% BSFL: Diet containing 20% Black Soldier Fly Larvae meal as a replacement for soybean meal. 40% BSFL: Diet containing 40% Black Soldier Fly Larvae meal as a replacement for soybean meal. 60% BSFL: Diet containing 60% Black Soldier Fly Larvae meal as a replacement for soybean meal.

**Table 3:** Nutrient Composition of Black Soldier Fly Larvae (as-fed basis).

Nutrient	Value
Moisture (%)	3.67
Crude Protein (%)	41.4
Crude Fat (%)	31.36
Crude Fiber (%)	6.54
Ash (%)	7.53
Calcium (%)	1.77
Phosphorus (%)	0.8
Ca:P Ratio	2.21
Potassium (%)	1.14
Magnesium (%)	0.31
Sodium (%)	0.13
Lauric Acid (%)	11
Linoleic Acid (%)	5.74
Linolenic Acid (%)	0.46
Lysine (%)	2.27
Methionine (%)	0.54
TMEn (kcal/kg)	4349

**Table 4:** Effect of Replacing Soybean Meal with Varying Levels of Black Soldier Fly Larvae (BSFL) on Broiler Growth Performance.

Items	BSFL Levels				SEM	*p*-Values	
	Control	20%	40%	60%		Lin.	Quad.
Body weight, g							
10 days	215.87	215.97	217.01	218.10	0.735	0.026	0.506
21 days	869.58	809.48	774.69	743.33	9.695	<0.001	0.149
42 days	3089.79	2877.50	2706.67	2573.44	52.717	<0.001	0.460
Daily body weight gain, g							
10–21 days	65.37	59.35	55.77	52.52	0.945	<0.001	0.153
21–42 days	105.72	98.48	92.00	87.15	2.269	<0.001	0.602
10–42 days	92.71	85.86	80.31	75.98	1.695	<0.001	0.464
Daily feed intake, g							
10–21 days	84.98	87.23	86.33	79.92	1.828	0.059	0.025
21–42 days	164.64	158.14	156.44	157.27	2.681	0.057	0.183
10–42 days	138.94	135.27	133.82	132.32	2.028	0.026	0.597
Feed conversion ratio							
10–21 days	1.30	1.47	1.55	1.53	0.039	0.002	0.018
21–42 days	1.56	1.61	1.70	1.81	0.031	<0.001	0.327
10–42 days	1.50	1.57	1.69	1.75	0.024	<0.001	0.971

Control: Basal diet without Black Soldier Fly Larvae meal. 20% BSFL: Diet containing 20% Black Soldier Fly Larvae meal as a replacement for soybean meal. 40% BSFL: Diet containing 40% Black Soldier Fly Larvae meal as a replacement for soybean meal. 60% BSFL: Diet containing 60% Black Soldier Fly Larvae meal as a replacement for soybean meal. SEM: standard error of the means. Lin: Linear responses to dietary inclusion of Black Soldier Fly Larvae meal levels. Quad: Quadratic responses to dietary inclusion of Black Soldier Fly Larvae meal levels.

**Table 5:** Effect of Replacing Soybean Meal with Varying Levels of Black Soldier Fly Larvae (BSFL) on Broiler Carcass Traits and Meat Quality.

Items	BSFL Levels				SEM	*p*-Values	
	Control	20%	40%	60%		Lin.	Quad.
Carcass traits							
Dressing%	64.77	63.30	61.17	62.44	0.775	0.014	0.088
Abdominal fat%	1.09	1.13	0.84	0.95	0.119	0.179	0.765
Liver%	1.85	1.68	1.63	1.74	0.098	0.383	0.153
Gizzard%	1.88	2.31	2.50	2.48	0.095	<0.001	0.025
Heart%	0.52	0.49	0.49	0.50	0.022	0.576	0.454
Spleen%	0.11	0.10	0.10	0.10	0.006	0.403	0.641
Pancreas%	0.19	0.18	0.18	0.19	0.012	0.598	0.402
Small intestine%	3.77	3.73	4.05	3.95	0.189	0.315	0.875
Cecum%	0.57	0.65	0.65	0.63	0.055	0.507	0.370
Meat quality							
L* (Lightness)	52.42	53.02	50.71	51.10	1.022	0.181	0.921
a* (Redness)	3.46	3.60	4.00	3.84	0.516	0.510	0.770
b* (Yellowness)	9.11	9.61	9.74	7.72	0.658	0.181	0.066

Control: Basal diet without Black Soldier Fly Larvae meal. 20% BSFL: Diet containing 20% Black Soldier Fly Larvae meal as a replacement for soybean meal. 40% BSFL: Diet containing 40% Black Soldier Fly Larvae meal as a replacement for soybean meal. 60% BSFL: Diet containing 60% Black Soldier Fly Larvae meal as a replacement for soybean meal. SEM: standard error of the means. Lin: Linear responses to dietary inclusion of Black Soldier Fly Larvae meal levels. Quad: Quadratic responses to dietary inclusion of Black Soldier Fly Larvae meal levels.

## Data Availability

The original contributions presented in this study are included in the article. Further inquiries can be directed to the corresponding author.

## Abbreviations

The following abbreviations are used in this manuscript:

AAFCO	Association of American Feed Control Officials
ALP	Alkaline phosphatase
AST	Aspartate aminotransferase
BSFL	Black soldier fly larvae
BUN	Blood urea nitrogen
Ca	Calcium
CPK	Creatine phosphokinase
DEB	Dietary electrolyte balance
FCR	Feed conversion ratio
GGT	Gamma-glutamyl transferase
GLM	General Linear Model
K	Potassium
L*	Lightness (meat color parameter)
a*	Redness (meat color parameter)
b*	Yellowness (meat color parameter)
Na	Sodium
P	Phosphorus
PVAMU	Prairie View A&M University
SBM	Soybean meal
SEM	Standard error of the mean
TMEn	Nitrogen-Corrected True Metabolizable Energy
USDA	United States Department of Agriculture
